# Elucidation of the key odorants contributing to the distinctive honey and sweet potato-like aroma in Wuyi black tea

**DOI:** 10.1016/j.fochx.2026.104022

**Published:** 2026-05-22

**Authors:** Zhichao Lin, Mengzhen Xia, Li Niu, Guohe Chen, Lianqing Wang, Jianan Huang, Zhonghua Liu, Chao Wang

**Affiliations:** a Key Laboratory of Tea Science of Ministry of Education, Hunan Agricultural University, Changsha 410128, China; b National Research Center of Engineering and Technology for Utilization of Botanical Functional Ingredients, Changsha 410128, China; c State Key Laboratory of Tea Plant Germplasm Innovation and Resource Utilization, Hunan Agricultural University, Changsha 410128, China; d Key Laboratory for Evaluation and Utilization of Gene Resources of Horticultural Crops, Ministry of Agriculture and Rural Affairs of China, Hunan Agricultural University, Changsha 410128, China; e Tea Cultivar Innovation Center, Yuelushan Laboratory, Changsha 410128, China

**Keywords:** Wuyi black tea, Honey aroma, Sweet potato-like aroma, GC × GC-TOFMS, Molecular docking-MD simulations

## Abstract

Honey and sweet potato-like aroma Wuyi black tea (WYBT) is highly favored by consumers for its warm and sweet flavor profile. However, the key odor-active compounds underlying this characteristic aroma have remained inadequately characterized. This study systematically characterized the unique honey and sweet potato-like aroma profile of WYBT through molecular sensory science approaches, leading to the identification of 19 key aroma-active compounds. Aroma recombination and omission experiments further established that 2-amylfuran, *α*-ionone, *β*-cyclocitral, *β*-damascenone, and benzeneacetaldehyde play central roles in constituting the characteristic aroma profile. Molecular docking revealed hydrogen-bonding and hydrophobic interactions between these five key compounds and olfactory receptors, and molecular dynamics (MD) simulation further confirmed the stability of these bindings. This study identifies the key odor-active compounds underlying the distinctive honey and sweet-potato-like aroma of WYBT, providing a theoretical basis for quality standardization and process optimization of this unique tea variety.

## Highlights

• Multiple compounds jointly present the honey and sweet potato like-aroma of tea.
• Nineteen compounds were identified as key aroma-active compounds.
• Five key compounds were identified as most important for characteristic aromas.
• Molecular dynamics simulation validate the binding stability of key components.

## 1 Introduction

Tea is one of the most widely consumed beverages worldwide and is valued not only for its flavor but also for its diverse bioactive properties, including antioxidant, anti-radiation, antibacterial, and anti-inflammatory activities (Peng et al., 2022). The production of black tea, a fully fermented tea, involves sequential steps of withering, rolling, fermentation, and drying (Chen et al., 2023). Throughout these processing stages, volatile components undergo complex chemical transformations that ultimately impart the characteristic aroma of black tea. Wuyi black tea (WYBT), a geographical indication product from Fujian Province, is distinguished by its local cultivar, *Camellia sinensis* var. *sinensis cv. Bohea*, which is characterized by strong cold tolerance and abundant aroma precursors, thereby contributing to its superior quality (Su et al., 2025). The aroma profile of black tea is a key quality determinant and a major driver of consumer preference and market success (Zhou et al., 2025). With increasing consumer demand for specific aroma styles, such as honey-like and sweet-potato-like notes, the diversification of black tea products has accelerated. Nevertheless, systematic research on this honey and sweet potato like aroma, an important sensory characteristic of this tea, remain limited.

Molecular sensory analysis has enabled the identification of characteristic aroma profiles in black tea. Among them, headspace solid-phase microextraction (HS-SPME) combined with comprehensive two-dimensional gas chromatography–time-of-flight mass spectrometry (GC × GC-TOFMS) and gas chromatography-olfactometry (GC-O) is particularly effective for characterizing low-molecular-weight, highly volatile compounds (Ouyang et al., 2025; Zhang et al., 2025). GC × GC-O-TOFMS analysis combined with aroma recombination and omission experiments identified four critical compounds that impart the minty aroma to Rucheng Baimaocha: (*E,Z*)-2,6-nonadienal, methyl salicylate, methyl geranate, and (*E*)-2-nonenal (Ouyang et al., 2025). Likewise, the characteristic honey-like aroma of Zunyi black tea was attributed to key contributors by key contributors including geraniol, 2-phenyl-2-butenal, furaneol, cinnamaldehyde, ethyl cinnamate, and 2-pentanone, as determined by GC-O-MS and OAV analysis (Sun et al., 2024). Despite progress in understanding black tea aromas, the key compounds responsible for the honey and sweet potato-like profile remain unconfirmed.

At present, the molecular basis of how aroma-active compounds are perceived after being identified through molecular sensory analysis is still not fully understood. Therefore, Molecular docking and molecular dynamics (MD) simulations have therefore been increasingly used to investigate the binding interactions between odorants and olfactory receptors. Molecular docking is a computational method that simulates the interaction between a ligand and a receptor protein to predict their binding mode and affinity (Zhu et al., 2025). However, binding energy derived from molecular docking is an imperfect predictor of perceived sensory enhancement (Hu et al., 2025). MD simulations reveal the dynamic behavior of complexes, overcoming the limitations of static models by quantifying interaction stability and identifying key energy determinants (Zhang et al., 2025). Together, molecular docking and MD simulations offer efficient prediction and three-dimensional structural insight into odorant-receptor interactions in food systems. For honey-flavored black tea, molecular docking demonstrated spontaneous binding of key odorants (*β*-damascenone, phenylacetaldehyde, linalool oxide I) to olfactory receptors, while MD simulations confirmed the stability of these complexes (Zhang et al., 2025). Thus, the combination of molecular docking and MD simulation provides a useful framework for revealing the potential binding mechanisms of aroma compounds with olfactory receptors at the atomic level.

Despite the successful application of molecular sensory approaches in other tea types, the key odorants and receptor-interaction mechanisms underlying the distinctive honey and sweet potato-like aroma of WYBT remain insufficiently understood. Therefore, the objectives of this study were: (1) to identify the key odor-active compounds responsible for the characteristic honey and sweet potato-like aroma of WYBT by integrating sensory evaluation, GC × GC-O-TOFMS analysis, and aroma recombination/omission experiments; and (2) to further explore the potential molecular recognition mechanisms of the decisive odorants using molecular docking and MD simulations. By combining sensory validation with computational interpretation, this study provides a more integrated understanding of the chemical basis and perceptual mechanism of this characteristic aroma profile.

## 2 Materials and methods

### 2.1 Tea samples

A total of forty-seven WYBT samples, all produced in spring 2024, were obtained from Fujian Wuyishan National Nature Reserve Lapsang Tea Industry Co., Ltd., located in the Tongmuguan area of the Wuyi Mountain National Nature Reserve, Fujian Province. All tea samples were processed according to traditional black tea manufacturing procedures. Before instrumental analysis, a preliminary sensory screening was carried out to identify samples that best represented the characteristic honey- and sweet potato-like aroma style of WYBT. The screening was performed by trained assessors following the same standardized sensory conditions and scoring principles adopted in the subsequent QDA analysis. In this step, the assessors evaluated the prominence, typicality, and consistency of the target aroma attributes across all 47 samples. Based on the consensus sensory results, six samples exhibiting the most representative and pronounced honey- and sweet potato-like aroma characteristics were selected and designated as WYBT-1 to WYBT-6. These six samples were subsequently used as representative materials for the construction of the characteristic aroma profile and for further volatile, sensory, and molecular analyses. The selected samples were then ground, passed through a 2.0 mm mesh sieve, individually sealed in bags, and stored under refrigerated conditions until use.

### 2.2 Chemicals

Ethyl decanoate and n-alkanes (C_7_-C_30_) were purchased from Sigma-Aldrich (Shanghai, China). Linalool, *β*-cyclocitral, 2-amylfuran, safranal, and nerol were purchased from J&K Scientific (Beijing, China); *β*-Damascenone, *α*-ionone, nonanal, *d*-limonene; (*E*)-nerolidol, and decanal were purchased from Aladdin (Shanghai, China); 1-Octen-3-ol, methyl salicylate, and linalool oxide I were purchased from Sigma-Aldrich (Shanghai, China); 2-Phenyl-2-butenal and longifolene were purchased from Macklin Biochem (Shanghai, China); Benzeneacetaldehyde was purchased from Thermo Fisher Scientific (Shanghai, China); (*E*)-*β*-Ocimene was purchased from HongYe Biotech (Shanghai, China); Dimethyl trisulfide was purchased from TMstandard (Jiangsu, China).

### 2.3 Sensory evaluation

Sensory evaluation was conducted with the approval of the Hunan Agricultural University Ethics Committee (Approval No: 2023–138, Changsha, China). The evaluation panel is composed of 12 experienced evaluators from Hunan Agricultural University (including 7 males and 5 females, aged between 25 and 30). Sensory evaluations of tea characteristic aromas were conducted in a standardized sensory evaluation room. Prior to formal testing, each assessor underwent a minimum of 90 h of specialized training to ensure accurate identification and quantification of various aroma attributes. The evaluation was carried out in accordance with the Chinese National Standard (GB/T 23776–2018). Specifically, 3 g of each tea sample was weighed and infused in 150 mL of boiling water for 5 min. The infusion was then filtered to remove the leaves before evaluation. Quantitative Descriptive Analysis (QDA) was performed using a 10-point intensity scale, where 0 indicated “not perceived”, 5 represented “medium intensity”, and 10 denoted “very strong”. Assessors selected five aroma descriptors that best characterized each sample. The final score for each sensory attribute was determined by averaging the ratings from the 12 panelists. The five aroma descriptors, namely sweet potato-like, honey-like, floral, fruity, and woody, were determined based on preliminary sensory screening of the WYBT samples, panel consensus among trained assessors, and reference to descriptors commonly reported in black tea aroma studies. Each attribute score was expressed as the mean value of the 12 assessors. To minimize evaluation bias, all samples were assessed under the same experimental conditions and using the same scoring criteria. All participants were informed of the purpose, procedures, and use of the sensory evaluation data before the sensory tests. Written informed consent was obtained from all participants prior to their participation. Participation was voluntary, and all sensory data were anonymized and reported only in aggregate form. No personal identifying information of the panelists was disclosed in the manuscript or supplementary materials.

### 2.4 Extraction of volatile compounds using the HS-SPME

The HS-SPME procedure was performed following an optimized method previously established by our research team (Ouyang et al., 2024). Briefly, 0.500 g of powdered tea sample was accurately weighed and transferred into a 20 mL headspace vial. The vial was then placed in the autosampler tray of a CTC system, which automatically added 10 μL of ethyl decanoate (8.63 mg/L) as the internal standard and 5 mL of boiling water. Subsequently, the vial was incubated at 60 °C with agitation at 600 rpm for 10 min to allow the sample to reach equilibrium. A 50/30 μm DVB/CAR/PDMS fiber was then exposed to the headspace for 30 min under the same temperature and agitation conditions. Finally, the fiber was thermally desorbed in the GC inlet at 250 °C for 10 min.

### 2.5 GC × GC-O-TOFMS analysis

Volatile compounds were analyzed using comprehensive two-dimensional gas chromatography coupled with time-of-flight mass spectrometry, GC × GC-TOFMS, equipped with an olfactory detection port. The system was fitted with an HP-5MS column for the first dimension and a DB-17MS column for the second dimension, both housed in a single oven. The temperature program was identical for both dimensions. The oven temperature was initially set at 40 °C for 1 min, then raised at 4 °C/min to 180 °C and further increased at 20 °C/min to 250 °C, where it was held for 1 min. A solid-state modulator, SSM 1800, was used with a modulation period of 4 s. The mass spectrometer was operated in electron ionization, EI, mode at 70 eV, with the ion source and quadrupole temperatures maintained at 200 °C and 150 °C, respectively. Mass spectra were acquired over an *m*/*z* range of 45 to 500. The retention index, RI, was calculated using an n-alkane series (Ouyang et al., 2025).

In the two dimensional mode, the effluent from the first dimension column was mixed with helium and subsequently divided into two equal streams. One stream was directed to the olfactory detection port, ODP, and the other to the mass detector. Olfactory analysis was conducted by three trained sensory evaluators. The evaluators recorded the retention time, aroma characteristics, and aroma intensity of each perceived odorant using a scale from 0 to 10, where 0 indicated no odor, 1 weak intensity, 5 moderate intensity, and 10 strong intensity. The reported intensity of each odorant was expressed as the mean value of the ratings provided by the three evaluators (Ouyang et al., 2024).

### 2.6 Identification and quantification of volatile compounds

The GC × GC-TOFMS data were processed using Canvas software (version 1.0.0.25117). Volatile compounds were identified by comparing their mass spectra with the NIST 20 database. RI were calibrated using a C₇–C_30_ n-alkane series. Volatile compounds were confirmed when they met all of the following criteria: forward match factor > 700, reverse match factor > 800, and RI deviation<30. Statistical comparison tools within the Canvas platform were applied to align and compare peak tables across samples. Relative concentrations of volatile compounds were quantified using the internal standard method with the following formula:

$$C_{i}\left(\mu g/\mathrm{kg}\right)=\frac{\mathrm{Ratio}\times 10\mathrm{ul}\times 8.63\;\mathrm{mg}/L}{M}$$

Note: *Ratio* is the ratio of the peak area of the compound to the peak area of the internal standard; *Ci* is the concentration of each odor compound (μg/kg); *M* is the mass of the tea sample, 0.5 g. Each sample is tested three times.

### 2.7 Calculation of ROAV and ACI

The contribution of volatile compounds to the overall aroma was evaluated using ROAV and aroma character impact (ACI).The ROAV was calculated as the ratio of a compound's concentration to its odor threshold in water (Zheng et al., 2025). Compounds with an ROAV greater than 1 were considered potential key aroma contributors. The ACI was used to further quantify the relative impact of each aroma-active compound (Zhang et al., 2025). The formulas for ROAV and ACI are as follows:

$$\text{ROAV}=\frac{\mathrm{Ci}}{\mathrm{Ti}}$$

$$\mathrm{ACI}=\frac{\text{ROAV}}{\sum_{K}\mathrm{ROA}V_{K}}$$

Note: *Ci* (μg/kg) is the relative content of odor compounds, *Ti* (μg/kg) is the threshold for odor compounds, $\sum_{K}\mathrm{ROA}V_{K}$and is the sum of ROAVs for all volatile compounds.

### 2.8 Aroma recombination and omission

Aroma recombination and omission experiments were performed to validate the role of the identified odorants. First, a recombinant model was prepared by dissolving the key aroma-active compounds, selected based on GC-O and ROAV results, in the blank matrix at their actual quantified concentrations. Panelists then performed a sensory evaluation of the overall aroma of this complete recombinant model. For the omission experiment, a series of models were prepared, each lacking a single target odorant. These models were compared against the complete recombinant using a triangle test (3-alternative forced choice, 3-AFC) as a compulsory procedure. A panel of twelve assessors performed each comparison in triplicate. The significance of the perceptual difference was determined based on the number of correct identifications: 8 correct (*p* < 0.05), 9 correct (*p* < 0.01), and 10 correct (*p* < 0.001) (Ouyang et al., 2025).

### 2.9 Molecular docking

The three-dimensional structures of key volatile compounds associated with the honey-sweet potato aroma in WYBT, including *β*-damascenone, benzeneacetaldehyde, *α*-ionone, *β*-cyclocitral, and 2-amylfuran were retrieved in SDF ligand format from the PubChem database (https://pubchem.ncbi.nlm.nih.gov/). These files were converted to PDB format using Openbabel. OR1A1 (UniProt ID: Q9P1Q5)and OR1D2 (UniProt ID: P34982) were selected as representative human olfactory receptor models because they have been frequently used in previous tea-aroma-related molecular docking studies and are considered suitable for probing the interaction patterns between key tea odorants and olfactory receptors (Chen et al., 2026; Chen et al., 2025; Ji et al., 2025). Their three-dimensional structures were downloaded from the UniProt database (https://www.uniprot.org/) in PDB format. Molecular docking was performed using AutoDock Vina 1.2.5 (Scripps Research, USA). Receptor proteins were prepared by removing water molecules and adding hydrogen atoms, then saved in PDBQT format. The resulting receptor and ligand PDBQT files were used as inputs for AutoDock Vina. Docking results were analyzed and visualized using PyMOL 2.5.5 (DeLano Scientific, LLC).

### 2.10 Molecular dynamics simulation

MD simulations were performed using GROMACS 2020.6 with the AMBER99SB force field and the SPC water model. Long-range electrostatic interactions were treated with the Particle Mesh Ewald (PME) method. The system was first equilibrated for 100 ps in the NVT ensemble, followed by 100 ps in the NPT ensemble at 300 K. Production simulations were conducted for 100 ns with a cutoff distance of 1.2 nm. Post-simulation analysis included calculation of the radius of gyration (Rg), root mean square deviation (RMSD), root mean square fluctuation (RMSF), and solvent accessible surface area (SASA) using GROMACS utilities. Binding free energies (ΔGbind) were calculated using the MM/PBSA approach.

### 2.11 Statistical analysis

All experiments were performed in triplicate, and the results are presented as mean values± standard deviation. Volatile compounds data analysis was carried out using Excel 2021 software. Percentage stacked charts，pie chart, and radar charts were generated using Origin 2024 (OriginLab Corporation, USA). Correlation Network and venn diagram were performed using the OmicStudio tools at https://www.omicstudio.cn/tool.

## 3 Results

### 3.1 Quantitative descriptive analysis of WYBT

**Fig. 1:**
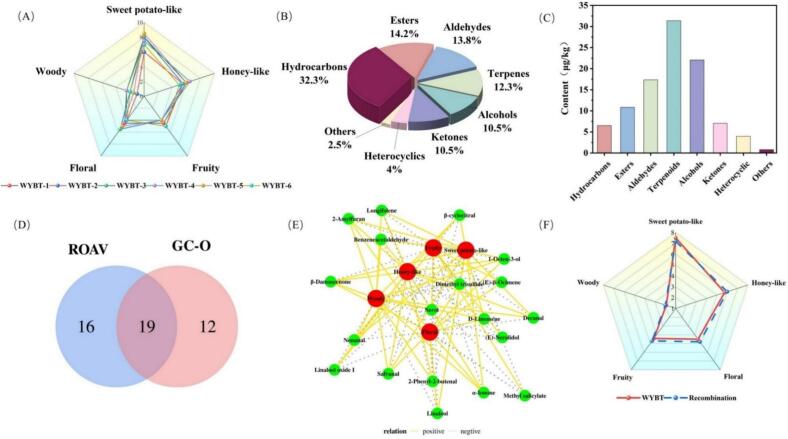
(A) QDA radar map of WYBTs with honey and sweet potato-like aroma； (B) Percentages Classification of 326 aroma compounds identified in WYBTs with honey and sweet potato-like aroma; (C) relative percentage concentrations of characteristic volatile compounds in WYBTs; (D) Venn diagram of aroma compounds identified in WYBTs; (E) Pearson correlation analysis between QDA and key aroma compounds; (F) Aroma profiles analyses of WYBT and the fully aroma recombinant.

QDA was performed to characterize the sensory profiles of the selected WYBT samples, as aroma is a key determinant of black tea quality and consumer preference. Based on preliminary screening, panel consensus, and literature support, five representative aroma descriptors were selected, including floral, fruity, honey-like, sweet-potato-like, and woody notes. As shown in Fig. 1A, the six selected WYBT samples shared a broadly comparable sensory orientation, with sweet-potato-like aroma as the dominant attribute and honey-like aroma as the secondary characteristic note. Floral and fruity notes were expressed at moderate levels, whereas woody aroma remained relatively weak. Although the selected samples were not identical in sensory composition, they exhibited a common target aroma style dominated by honey- and sweet potato-like features.

The sensory correlation heatmap (Fig. S1) provided additional insight into the internal structure of the sensory dataset. Fruity and honey-like attributes showed a significant positive correlation, while honey-like aroma was also positively associated with sweet-potato-like notes, indicating that these descriptors may act cooperatively in shaping the characteristic aroma style of WYBT. Sweet-potato-like aroma further showed a positive relationship with woody notes, suggesting the presence of a supporting background nuance. In contrast, floral aroma exhibited an overall negative trend with honey-like and sweet-potato-like attributes. These results indicate that the characteristic aroma of WYBT is shaped by a coordinated sensory balance among multiple descriptors, thereby supporting the subsequent investigation of its underlying odor-active compounds.

### 3.2 Characterization of volatile compounds in honey and sweet potato-like WYBT

Based on the QDA results, which showed that sweet-potato-like and honey-like notes were the dominant sensory features of the selected WYBT samples, volatile analysis was subsequently performed to identify the chemical basis underlying this characteristic aroma profile. HS-SPME-GC × GC-TOFMS was employed to analyze the volatile composition of selected samples exhibiting this characteristic profile. For accurate assessment of key volatiles contributing to the honey and sweet potato-like aroma, the average concentrations of detected compounds were calculated across six representative WYBT samples to establish the characteristic aroma profile (Table S1). A total of 323 volatile compounds were identified across the six WYBT samples. Hydrocarbons (105 compounds, 32.3%) constituted the most abundant class, followed by esters (47, 14.5%), aldehydes (45, 13.5%), terpenes (40, 12.3%), alcohols (34, 10.5%), and ketones (34, 10.5%), which together form the main aromatic components in WYBT (Fig. 1B). The diversity and abundance of these compound classes provide a substantial chemical foundation for the development of WYBT's characteristic honey and sweet potato-like aroma.

A more detailed analysis of the volatile composition demonstrated. The relative content of volatile classes in honey and sweet potato-like WYBT, as shown in Fig. 1C, was dominated by terpenes (31.38%), followed by alcohols (22.08%), aldehydes (17.36%), and esters (10.85%). Specifically, the following volatile compounds were present at notably higher concentrations compared to others: linalool (828.34 μg/kg), nerol (780.11 μg/kg), linalool oxide I (731.69 μg/kg), methyl salicylate (435.71 μg/kg), benzeneacetaldehyde (324.42 μg/kg), and benzeneethanol (319.20 μg/kg). The combination of these compounds collectively shapes the unique and complex flavor profile of tea. Although hundreds of volatile compounds could be detected in food matrices, only a limited number of key odorants significantly contribute to the overall aroma. This is because the perceived aroma depends not only on the concentrations of aroma compounds, but also on their odor thresholds. Thus, ROAV calculation was employed to identify the key aroma compounds in this study.

The ROAV is commonly employed in tea aroma studies to quantify the relative impact of each compound within the overall flavor profile (Chen et al., 2025). Based on the quantitative data of volatile compounds obtained by GC × GC-TOFMS, the ROAV values were calculated using odor thresholds from published literature or relevant databases (Table 1). Applying a threshold of ROAV>1, 35 key aroma compounds were identified. Notably, thirteen compounds exhibited ROAV values greater than 10, collectively constituting 85.33% of the total aroma characteristic index (ACI). Seven key compounds, including linalool, *β*-damascenone, benzeneacetaldehyde, *α*-ionone, *β*-cyclocitral, 1-octen-3-ol, and 2-amylfuran, demonstrated high ROAV values and were proposed as the primary contributors to the honey and sweet potato-like aroma of WYBT. While ROAV analysis provides relatively objective results, significant controversies persist regarding its threshold determination methodology and outcomes. Consequently, integrating OAV with GC-O approaches becomes essential for accurately characterizing the sample's distinctive flavor components.

**Table 1:** Aroma compounds with an ROAV >1 in WYBTs with honey and sweet potato-like aroma.

NO.	Aroma active compounds	CAS^a^	RI^b^	NIST RI	Odor description^c^	OTs^d^ (μg/kg)	Concentration^e^ (μg/kg)	ROAV	ACI
1	Linalool	78–70-6	1093	1099-S	Floral, sweet, grape-like, woody	6	828.34 ± 317.89	138.06	22.26%
2	*β*-Damascenone	23,726–93-4	1378	1386-S	Floral, sweet	0.43	37.56 ± 19.84	87.36	14.09%
3	Benzeneacetaldehyde	122–78-1	1038	1045-S	Sweet and fragrant honey	4	324.42 ± 91.19	81.11	13.08%
4	*α*-Ionone	127–41-3	1422	1426-S	Floral, violet-like, powdery, berry-like	0.4	25.18 ± 12.84	62.95	10.15%
5	*β*-cyclocitral	432–25-7	1214	1220-S	Herbal, rose-like, fruity	3	93.77 ± 60.97	31.26	5.04%
6	1-Octen-3-ol	3391-86-4	969	980-S	Earthy, green, oily, vegetative-like, fungal	1.5	40.99 ± 23.16	27.33	4.41%
7	2-Amylfuran	3777-69-3	982	993-S	Fruity, green, earthy beany with vegetable like	4.8	110.3 ± 55.08	22.98	3.71%
8	Safranal	116–26-7	1194	1201-S	Woody, spicy, medicinal, powdery, and herbal	3	57.36 ± 10.41	19.12	3.08%
9	Nerol	106–25-2	1247	1228-S	Fresh, citrus, floral, green, sweet, lemon-like	49	780.11 ± 272.37	15.92	2.57%
10	Nonanal	124–19-6	1097	1104-S	Floral, fatty, green, lemon-like	8	90.69 ± 85.89	11.34	1.83%
11	Methyl salicylate	119–36-8	1188	1192-S	Green	40	435.71 ± 119.1	10.89	1.76%
12	*β*-Ocimene	13,877–91-3	1040	1037-S	Citrus, herbal, spicy, sweet	6.7	72.77 ± 45.78	10.86	1.75%
13	Ethyl hexanoate	123–66-0	991	999-S	Fruity, pineapple-like, sweet, green, waxy	2.2	21.99 ± 15.77	10.00	1.61%
14	d-Limonene	5989–27-5	1020	1031-S	Fruity, lemon-like	10	77.73 ± 87.49	7.77	1.25%
15	Dehydro-ar-ionene	30,364–38-6	1348	1354-S	Licorice-like	2.5	19.41 ± 12.76	7.76	1.25%
16	Linalool oxide I	5989-33-3	1066	1074-S	Sweet, floral, creamy	100	731.69 ± 287.82	7.32	1.18%
17	(*E*)-2-Octenal	2548-87-0	1052	1060-S	Fresh, cucumber-like, fatty, green, herbal, leafy	3	20.33 ± 13.87	6.78	1.09%
18	Dimethyl trisulfide	3658-80-8	961	971-S	Bitter	2.68	17.34 ± 2.2	6.47	1.04%
19	Heptanal	111–71-7	894	901-S	Heavy, plant green odor, apricot-like, and nutty	3	17.98 ± 7.75	5.99	0.97%
20	Cedrol	77–53-2	1601	1600-S	Mild cedar wood-like aroma	1	5.91 ± 6.73	5.91	0.95%
21	*β*-Myrcene	123–35-3	982	991-S	Lemon, woody, floral	15	84.13 ± 32.3	5.61	0.90%
22	Hexanal	66–25-1	792	801-S	Grassy, green, fresh, fatty	19	104.35 ± 44.69	5.49	0.89%
23	*p*-Cymene	99–87-6	1017	1025-S	Oily, resin	5.01	24.66 ± 13.2	4.92	0.79%
24	(*E*)-Nerolidol	40,716–66-3	1555	1564-S	Floral, green, citrus, woody, waxy	10	48.74 ± 46.13	4.87	0.79%
25	2-Hexenal	505–57-7	844	851-S	Grassy, herbal	30	101.53 ± 85.76	3.38	0.55%
26	(*Z*)-Jasmone	488–10-8	1393	1395-S	Floral, woody, herbal, citrus, jasmine-like	7	21.65 ± 24.79	3.09	0.50%
27	2-Phenyl-2-butenal	4411-89-6	1267	1276-S	Green, vegetable-like, floral, cocoa-like	14	38.93 ± 24.72	2.78	0.45%
28	Longifolene	475–20-7	1403	1406-S	Woody	2	4.77 ± 1.26	2.38	0.38%
29	Decanal	112–31-2	1197	1206-S	Aldehyde-like, candle-like, fatty and citrus-like	30	55.82 ± 19.36	1.86	0.30%
30	3,7-Dimethyl-1,5,7-octatrien-3-ol	29,957–43-5	1097	1107-S	Fresh, floral, fruity	110	203.49 ± 116.66	1.85	0.30%
31	(*E*)-β-Ocimene	3779-61-1	1029	1049-S	Warm, floral, herbal, sweet	34	54.36 ± 26.89	1.60	0.26%
32	Benzyl alcohol	100–51-6	1027	1036-S	Floral, rose-like, phenolic, balsamic	100	147.06 ± 49.81	1.47	0.24%
33	(3*Z*)-3-Hexen-1-yl hexanoate	31,501–11-8	1372	1380-S	Fruity, waxy, green, fatty, winey	70	87.74 ± 31.78	1.25	0.20%
34	(*E*)-Neral	141–27-5	1262	1270-S	Lemon	32	39.86 ± 41.22	1.25	0.20%
35	(*E,E*)-3,5-Octadien-2-one	30,086–02-3	1064	1073-S	Creamy, fruity	100	114.62 ± 70.96	1.15	0.18%

^a^CAS, the published chemical abstracts service (CAS) of compounds in NIST 20 library. ^b^ RI, retention index; ^c^ The odor description eferred to the database (The Good Scents Company Information System, http://www.thegoodscentscompany.com/). ^d^ OTs: Odor thresholds in water. These values were sourced from published literature or relevant websites.

### 3.3 Identification of key aroma components in WYBT with honey and sweet potato-like aroma by GC-O

Olfactory technology is a well-established tool for screening aroma-active compounds, and commonly used for food flavor analysis (Ouyang et al., 2024). To characterize the aroma profile and intensity of volatile compounds in honey and sweet potato-like aroma of WYBT, six representative samples were blended in equal proportions and analyzed by GC × GC-O-TOFMS. A total of 31 aroma-active compounds were identified (Table 2). Among them, *α*-ionone (AI: 8.25; fruity, floral), benzeneacetaldehyde (AI: 8.00; sweet, honey), *β*-damascenone (AI: 7.50; floral, sweet), 2-amylfuran (AI: 7.13; fruity, beany), linalool (AI: 6.75; floral, sweet), and *β*-cyclocitral (AI: 6.50; fruity) were characterized by high aroma intensities. Together, these compounds significantly shape the characteristic honey and sweet potato like aroma of WYBT. It was worth noting that among the 34 aroma compounds detected via olfactometry, none were individually described as having a distinct honey and sweet potato-like aroma. This suggests that the distinctive sweet potato-like aroma in WYBT likely originates from the combinatorial effect of multiple aroma-active compounds working in concert, rather than being dominated by any single component, collectively contributing to its unique flavor quality.

**Table 2:** Aroma compounds identified by GC × GC-O in WYBTs with honey and sweet potato-like aroma.

No.	Volatile compounds	RT	CAS	Odor description	AI
1	Benzaldehyde	11.94	100-52-7	Fruity，nutty	5.50
2	Dimethyl trisulfide	12.28	3658-80-8	Corn	3.25
3	1-Octen-3-ol	12.61	3391-86-4	Fungal	5.75
4	6-Methyl-5-hepten-2-one	12.94	110-93-0	Fruity	6.13
5	2-Amylfuran	13.08	3777-69-3	Fruity，beany	7.13
6	(*E,E*)-2,4-Heptadienal	13.81	4313-3-5	Fatty	2.63
7	*α*-Terpinene	14.08	99-86-5	Fruity	2.88
8	d-Limonene	14.48	5989-27-5	Fruity	5.25
9	2,2,6-trimethylcyclohexanone	14.74	2408-37-9	Honey，fruity	4.63
10	(*E*)*-β*-Ocimene	14.81	3779-61-1	Floral, herbal, sweet	5.00
11	Benzeneacetaldehyde	15.08	122-78-1	Sweet，honey	8.00
12	Linalool oxide I	16.14	5989-33-3	Sweet, floral,	6.38
13	Linalool	17.14	78-70-6	Floral, sweet	6.75
14	Nonanal	17.29	124-19-6	Floral, fatty，fruity	6.25
15	Benzeneethanol	17.61	60-12-8	Honey，floral	6.00
16	Linalool oxide IV	19.68	39028-58-5	Floral, honey	4.63
17	*α*-Terpineol	20.48	98-55-5	Floral	3.75
18	Methyl salicylate	20.61	119-36-8	Green	6.25
19	Safranal	20.81	116-26-7	Woody，herbal	5.25
20	Decanal	20.94	112-31-2	Fatty，fruity	6.25
21	(*E,E*)-2,4-Nonadienal	21.28	5910-87-2	Fatty	5.13
22	*β*-Cyclocitral	21.54	432-25-7	fruity	6.50
23	(3*Z*)-3-Hexenyl 2-methylbutanoate	21.88	53398-85-9	Fruity	5.00
24	(*Z*)-3,7-Dimethyl-2,6-octadienal	22.21	106-26-3	Fruity	2.50
25	Nerol	22.68	106-25-2	Floral, sweet，fruity	5.38
26	2-Phenyl-2-butenal	23.34	4411-89-6	Foral, cocoa-like	4.13
27	Theaspirane	24.88	36431-72-8	Honey	3.75
28	*β*-Damascenone	27.08	23726-93-4	Floral, sweet	7.50
29	Longifolene	27.88	475-20-7	Woody	3.63
30	*α*-Ionone	28.48	127-41-3	Fruity，floral	8.25
31	(*E*)-Nerolidol	32.54	40716-66-3	Floral，Fruity，woody	6.13

### 3.4 Odor-active compounds in WYBT with honey and sweet potato-like aroma

Based on the integration of ROAV>1 and GC × GC-O-TOFMS analysis, 19 key aroma compounds were identified as significant contributors to the honey and sweet potato-like aroma of WYBT, as summarized in Fig. 1D. These compounds include: linalool, *β*-damascenone, benzeneacetaldehyde, *α*-ionone, *β*-cyclocitral, 1-octen-3-ol, 2-amylfuran, safranal, nerol, nonanal, methyl salicylate, *d*-limonene, linalool oxide I, dimethyl trisulfide, (*E*)-nerolidol, 2-phenyl-2-butenal, longifolene, decanal, and (*E*)-*β*-ocimene. To more directly link sensory perception with chemical composition, Pearson correlation analysis was performed between the intensities of the five QDA aroma attributes and the concentrations of the 19 key odor-active compounds identified by ROAV and GC-O analyses Fig. 1E. This analysis provided a sensory–chemical bridge for interpreting which compounds were most closely associated with the dominant honey-like and sweet-potato-like attributes of WYBT. The analysis revealed that nine compounds, including linalool and α-ionone, exhibited positive correlations with floral aroma, while five volatiles such as (*E*)-nerolidol and nerol were positively associated with fruity notes. It was noteworthy that thirteen components exhibited positive correlations with sweet potato-like aroma, with key representatives being *β*-damascenone, benzeneacetaldehyde, *α*-ionone, *β*-cyclocitral, and 2-amylfuran. Similarly, multiple compounds demonstrated positive associations with honey-like aroma, particularly *β*-damascenone, benzeneacetaldehyde, and *α*-ionone. These results highlight that several key odorants contribute to multiple aromatic attributes, underscoring their synergistic role in defining the complex flavor profile of WYBT. Pearson correlation analysis quantifies the linear relationship between two variables. Typically, an absolute r value greater than 0.6 indicates a strong correlation (Chen et al., 2025). Notably, *α*-ionone and 2-amylfuran showed strong positive correlations with sweet potato-like aroma (*r* > 0.6, *p* < 0.05), and benzeneacetaldehyde exhibited similarly strong correlations with honey-like aroma. These robust statistical relationships highlight these specific compounds as pivotal contributors to the distinctive sweet-potato and honey aromatic characteristics that define WYBT's unique flavor profile.

### 3.5 Aroma recombination and omission experiments

Simulating recombination tests provides a robust approach to validate analytical measurements (Ouyang et al., 2025). A recombinant model of the honey and sweet potato-like aroma in WYBT was established based on the average concentrations of 19 selected aroma-active compounds identified across six WYBT samples. Twelve panelists conducted comparative sensory evaluations between the original WYBT blend and the recombinant model. As shown in Fig. 1F, no significant difference was found in the overall aroma profiles between the recombinant model and the natural tea samples. This result confirms that the aroma profile of WYBT was accurately replicated in the recombinant model, demonstrating the successful identification and characterization of all key odor-active compounds responsible for its honey and sweet potato-like aroma.

To investigate the individual contributions of aroma-active compounds to the overall aroma profile of WYBT, we conducted systematic aroma omission experiments. Twelve trained panelists evaluated the differences between the complete recombinant model and individual omission models using 3-AFC. As summarized in Table 3, fifteen key aroma compounds showed statistically significant differences (*p* < 0.05) when omitted: *β*-damascenone, benzeneacetaldehyde, *α*-ionone, *β*-cyclocitral, 2-amylfuran, *d*-limonene, linalool oxide I, (*E*)-nerolidol, linalool, nerol, nonanal, methyl salicylate, dimethyl trisulfide, decanal, and (*E*)-*β*-ocimene. Notably, five compound *β*-damascenone, benzeneacetaldehyde, *α*-ionone, *β*-cyclocitral, and 2-amylfuran demonstrated decisive roles in forming WYBT's characteristic honey and sweet potato-like aroma (*p* < 0.001). Linalool, *d*-limonene, linalool oxide I, dimethyl trisulfide (*E*)-nerolidol, decanal, and (*E*)-*β*-ocimene were identified as important contributors to the overall aroma profile (*p* ≤ 0.01). Significant differences (*p* < 0.05) were also observed when nerol, nonanal, and methyl salicylate were omitted from the recombinant model. Conversely, no significant differences were detected in the absence of 1-octen-3-ol, safranal, 2-phenyl-2-butenal, decanal, and (*E*)-*β*-ocimene (*p* > 0.05). This lack of perceived difference might be attributed to mutual masking effects among compounds, which reduces their individual sensory impact. In conclusion, *β*-damascenone, benzeneacetaldehyde, *α*-ionone, *β*-cyclocitral, and 2-amylfuran constitute the core compounds responsible for the distinctive honey and sweet potato-like aroma in WYBT. On the basis of this stepwise sensory and chemical screening, these five decisive compounds were subsequently subjected to molecular docking and MD simulations to provide a receptor-level interpretation of their potential roles in aroma perception.

**Table 3:** Triangle test results of omission experiments for key aroma active compounds in WYBT with honey and sweet potato-like aroma.

No.	Key aroma active compounds	Right of selection^a^	Significance^b^
1	Linalool	8	*
2	*β*-Damascenone	10	***
3	Benzeneacetaldehyde	11	***
4	*α*-Ionone	10	***
5	*β*-Cyclocitral	10	***
6	1-Octen-3-ol	6	=
7	2-Amylfuran	10	***
8	Safranal	5	=
9	Nerol	8	*
10	Nonanal	8	*
11	Methyl salicylate	8	*
12	d-Limonene	9	**
13	Linalool oxide I	9	**
14	Dimethyl trisulfide	8	*
15	(E)-Nerolidol	9	**
16	2-Phenyl-2-butenal	4	=
17	Longifolene	8	*
18	Decanal	6	=
19	(E)-β-Ocimene	4	=

a Number of correct judgments from 12 experts.

b Significance:“*” representing significant (*p* < 0.05); “**” representing significant (*p* ≤ 0.01); “***” representing significant (*p* ≤ 0.001)**.**

### 3.6 Molecular docking of key aroma compounds with olfactory receptors

Molecular docking can verify the function of key flavor compounds and elucidate the formation mechanisms of flavors mediated by small molecules (Zhang et al., 2025). The presence of broad-spectrum receptors facilitates exploration into how diverse aromatic compounds interact (Zhu et al., 2025). We selected OR1A1 and OR1D2 for this study due to their prominence and extensive application in modeling tea aroma compounds (Ma et al., 2025). Molecular docking was performed as a mechanistic extension of the sensory and analytical results rather than as a primary screening tool. Among the 323 volatile compounds identified, 19 odorants were retained as key contributors based on ROAV and GC-O results, and five of them were further confirmed as decisive odorants by omission tests. Therefore, *β*-damascenone, benzeneacetaldehyde, *α*-ionone, *β*-cyclocitral, and 2-amylfuran were selected for molecular docking and MD simulations as the final target ligands. The purpose of this analysis was to compare the interaction patterns of the final decisive odorants with model olfactory receptors and thereby provide a plausible molecular interpretation of their sensory importance in WYBT.

As shown in Table 4, despite variations in the binding sites, the consistently negative binding energies observed for all complexes indicate spontaneous binding between the olfactory receptors and aromatic compounds. Comparative analysis of the binding energies of the five key aroma compounds with the two olfactory receptors revealed that 2-amylfuran exhibited the weakest binding affinity to both receptors (−5.737 kcal/mol and − 5.464 kcal/mol), while *β*-damascenone showed the strongest binding affinity (−7.685 kcal/mol and − 7.920 kcal/mol).This was followed by α-ionone (−6.785 kcal/mol and − 7.741 kcal/mol), *β*-cyclocitral (−7.285 kcal/mol and − 6.987 kcal/mol), and benzeneacetaldehyde (−6.222 kcal/mol and − 6.303 kcal/mol). These findings demonstrate pronounced binding affinity and specific molecular interactions between these two olfactory receptors and the five key aroma compounds (Sun et al., 2024). Binding energy quantifies molecular interactions, with more negative values indicating stronger affinity and complex stability (Chen et al., 2025). Consequently, aroma compounds with high binding affinity preferentially occupy the finite sites on olfactory receptors. This molecular selectivity provides a mechanistic explanation for their distinct aroma profiles and detection thresholds.

**Table 4:** The docking results between ORs (OR1A1 and OR1D2) and ligands.

Ligand	Receptors	Binding energy (kcal/mol)	Hydrogen bonds	Hydrophobic interactions
2-Amylfuran	OR1A1	−5.737		MET-199, GLY-202, VAL-203, PHE-206, VAL-254, ILE-105, ILE-181
	OR1D2	−5.464		LEU-199, ILE-200, GLY-203, PHE-207, LEU-255
*α*-Ionone	OR1A1	−6.785	TYR-265	PRO-183, LYS-186, LEU-262, PRO-261
	OR1D2	−7.741		LEU-199, GLY-203, LEU-208, PHE-207, LEU-255, LEU-260
*β*-cyclocitral	OR1A1	−7.285	TYR-258	MET-104, ILE-181, ILE-105, GLY-202, VAL-203, PHE-206, VAL-254
	OR1D2	−6.987		PHE-207, LEU-255, LEU-260, LEU-199, GLY-203, LEU-208
*β*-Damascenone	OR1A1	−7.685		GLY-202, ILE-105, MET-104, LEU-107, PHE-73, GLY-108, VAL-254, PHE-206, VAL-203
	OR1D2	−7.920		LEU-260, LEU-208, PHE-207, GLY-203, LEU-255
Benzeneacetaldehyde	OR1A1	−6.222	ILE-105, ASN-109	MET-104, GLY-108, VAL-254, PHE-206, VAL-203, GLY-202
	OR1D2	−6.303	TYR-155	GLY-203, LEU-199, LEU-255, LEU-207

**Fig. 2:**
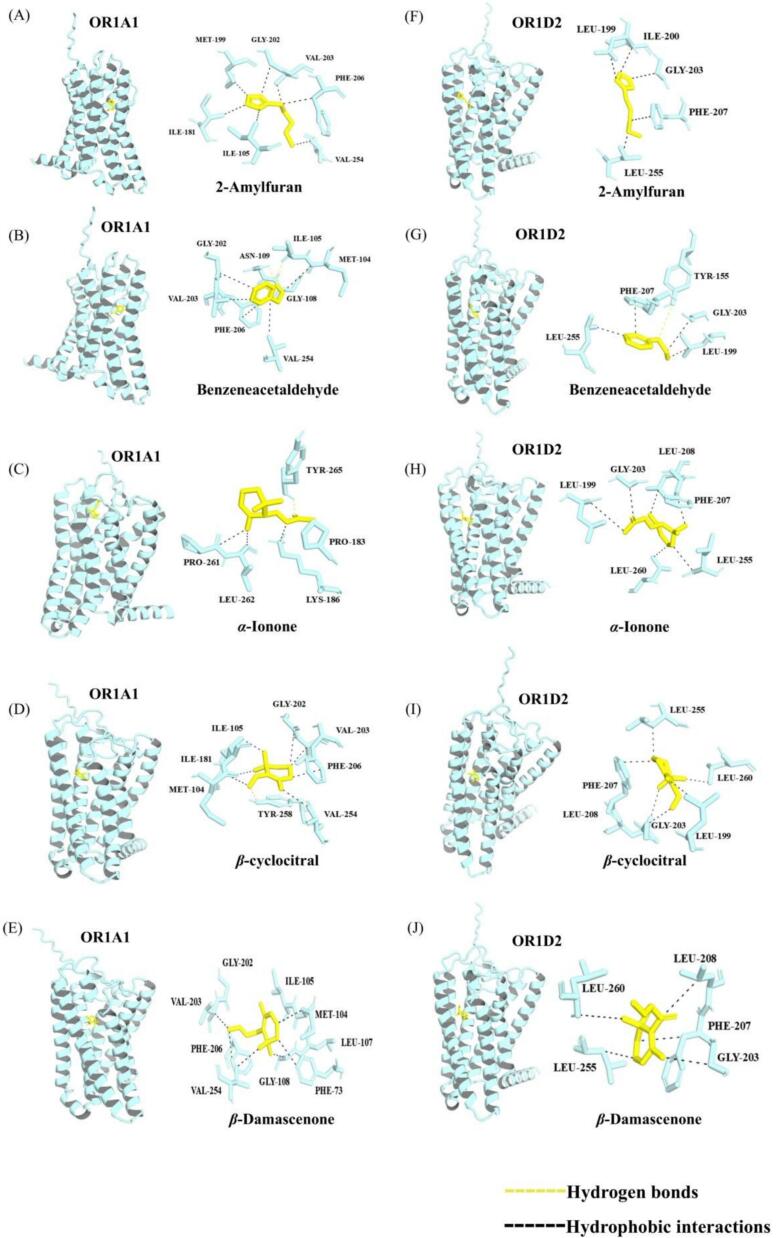
Key aroma compounds interacting with olfactory receptors. (A) Interaction of 2-amylfuran with the OR1A1; (B) Interaction of benzeneacetaldehyde with the OR1A1; (C) Interaction of *α*-ionone with the OR1A1. (D) Interaction of *β*-cyclocitral with the OR1A1. (E) Interaction of *β*-damascenone with the OR1A1. (F) Interaction of 2-amylfuran with the OR1D2. (G)Interaction of benzeneacetaldehyde with the OR1D2. (H) Interaction of *α*-ionone with the OR1D2. (I) Interaction of *β*-cyclocitral with the OR1D2. (J) Interaction of *β*-damascenone with the OR1D2.

Fig. 2 illustrates the molecular docking results of OR1A1 and OR1D2 with their respective ligands. The analysis reveals that hydrogen bonding and hydrophobic interactions play pivotal roles in the binding of aroma volatiles to olfactory receptors. Specifically, *α*-ionone formed a hydrogen bond with OR1A1 at residue TYR-265, while *β*-cyclocitral established a hydrogen bond at TYR-258 of the same receptor. Benzeneacetaldehyde demonstrated particularly versatile binding capability, forming hydrogen bonds with multiple residues across both receptors: ILE-105 and ASN-109 in one receptor, and TYR-155 in the other receptor. Hydrophobic amino acid residues create a hydrophobic pocket, promoting stable binding of odorant molecules (Chen et al., 2025). All five aroma compounds interact with multiple hydrophobic residues within two olfactory receptors. These structural insights demonstrate how specific amino acid residues create complementary hydrophobic environments that facilitate stable binding of diverse odorant molecules to olfactory receptors. In summary, the characteristic aroma components of honey and sweet potato-like of WYBT bind to olfactory receptors primarily via hydrophobic interactionsand and hydrogen bonds, which constitutes the molecular basis for perceiving its complex aroma profile. However, it is important to note that molecular docking relies on static receptor simulations and does not account for dynamic conformational changes, multi-receptor synergies, or the physiological conditions of the nasal environment, thereby limiting its ability to fully model the complexity of in vivo olfaction. Although molecular docking excels at the rapid identification and prediction of initial complex binding modes, molecular dynamics simulations are critical for characterizing the temporal stability and dynamic structural changes of these complexes.

### 3.7 Molecular dynamics simulation

**Fig. 3:**
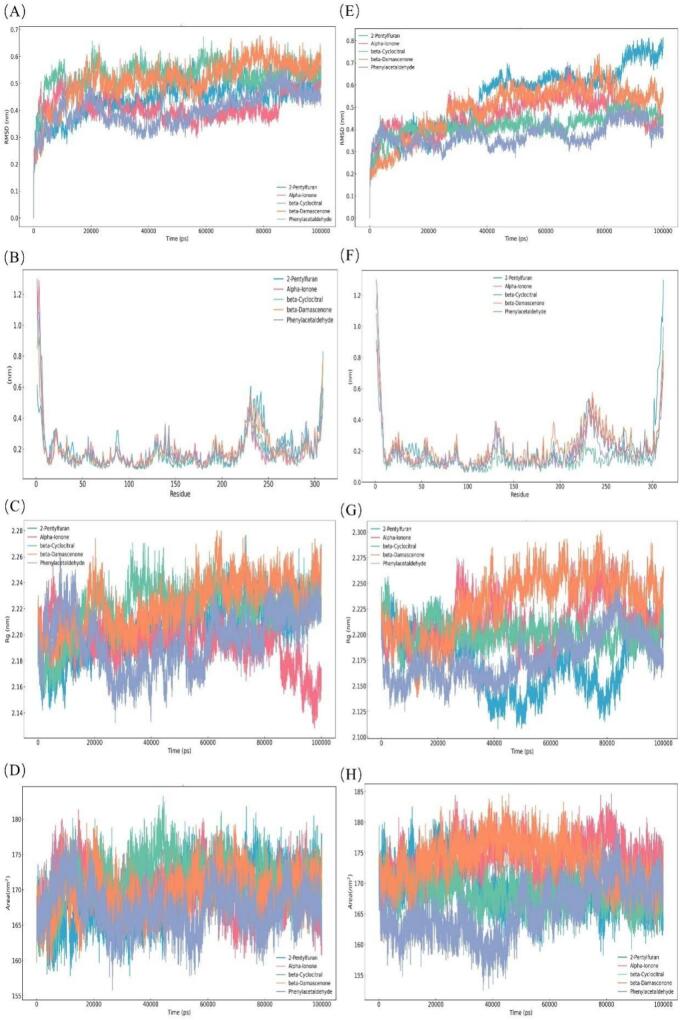
Molecular dynamics simulation analysis. (A) RMSD of five compounds and OR1A1; (B) RMSF of five compounds and OR1A1; (C) RG of five compounds and OR1A1; (D) SASA of five compounds and OR1A1; (E) RMSD of five compounds and OR1A1; (F) RMSF of five compounds and OR1A1; (G) RG of five compounds and OR1A1; (H) SASA of five compounds and OR1A1.

**Fig. 4:**
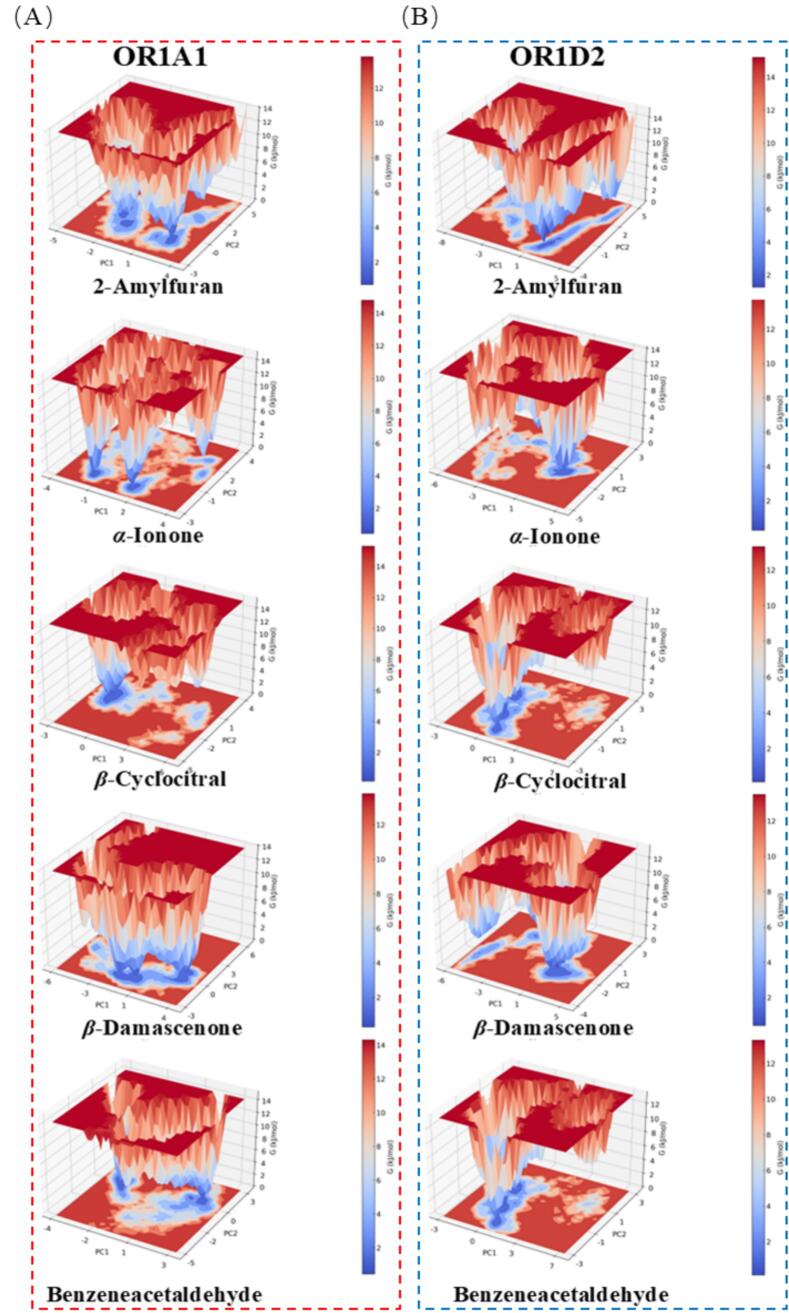
(A) Free energy landscape of the Ligand-OR1A1 complex; (B) Free energy landscape of the Ligand-OR1D2 complex.

To investigate the dynamic behavior of the molecular docking results, we conducted 100 ns MD simulations. Based on the molecular docking results, five key aroma compounds exhibiting strong binding affinities (2-amylfuran, *α*-ionone, *β*-cyclocitral, *β*-damascenone, and benzeneacetaldehyde) along with two olfactory receptors (OR1A1 and OR1D2) were selected for further investigation (Fig. 3). The stability of a protein-ligand complex is evaluated using the root mean square deviation (RMSD). Lower fluctuations and a plateau in the RMSD curve signify greater conformational stability of the complex (Zhang et al., 2025). The 10 complexes exhibited significant fluctuations during the initial simulation stage (0–20 ns), likely reflecting the ongoing establishment of interactions between the aromatic compounds and their receptor binding sites (Hu et al., 2025). As ligand-receptor contacts strengthened, the systems reached relative stability after approximately 50 ns, indicating equilibrium had been achieved with structurally stable complexes. Local flexibility was examined using root mean square fluctuation (RMSF) to assess residue-level displacements relative to the mean structure (Dai et al., 2024). The consistently low RMSF values confirmed stable binding of the aromatic compounds to the receptors. Additionally, the radius of gyration (Rg), a key indicator of structural compactness, fluctuated around 2.20 nm with minimal variation, demonstrating that these complexes maintained relatively dense configurations throughout the simulation (Tuo et al., 2024). Solvent Accessible Surface Area (SASA) includes hydrophobic and hydrophilic solvent accessible surface areas. The solvent-accessible surface area (SASA) values remained relatively stable, indicating no major structural alterations occurred during the simulation. The free energy landscape analysis was performed using principal components PC1 (x-axis) and PC2 (y-axis) against Gibbs free energy (z-axis) (Fig. 4). The results revealed that all 10 complexes formed one or more well-defined minimum energy basins, suggesting multiple stable binding modes rather than a single rigid conformation. The depth and distribution of these energy minima varied among receptors for the same ligand, indicating that receptors achieve selectivity through preferential stabilization of specific binding modes.

**Fig. 5:**
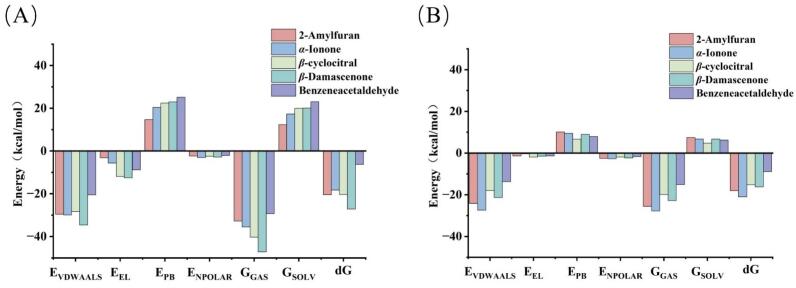
Statistical analysis of the MM/PBSA results for complexes. (A) Free energy of the ligand-OR1A1 complex; (B) Free energy of the ligand-OR1D2 complex.

MM/PBSA calculations (Fig. 5) further elucidated the interaction mechanisms, identifying van der Waals forces (ranging from −13.73 to −34.65 kcal/mol) as the dominant stabilizing contribution. This finding not only confirms the critical role of hydrophobic interactions in stabilizing these aromatic compound complexes but also aligns with previously reported computational studies on similar flavor-receptor systems (Zhang et al., 2025). The binding free energies of 2-amylfuran, *α*-ionone, *β*-cyclocitral, and *β*-damascenone with both OR1A1 and OR1D2 were relatively low, indicating strong binding affinity. Notably, benzeneacetaldehyde exhibited the highest binding free energies among all complexes: −6.34 ± 2.69 kcal/mol with OR1A1 and − 8.92 ± 1.93 kcal/mol with OR1D2, representing moderate binding strength. This result differs from its moderate affinity observed in the static molecular docking stage. Such discrepancies are expected because docking evaluates ligand–receptor compatibility in a fixed conformation, whereas MD/MM-PBSA incorporates receptor flexibility, solvent effects, and conformational entropy. The higher structural flexibility and larger conformational fluctuations of benzeneacetaldehyde during MD likely reduce its binding persistence, resulting in weaker free energies (Zhang et al., 2025). Therefore, further investigation is warranted to understand the binding stability between benzeneacetaldehyde and various olfactory receptors, aiming to identify the optimal receptor pairing with the most stable binding characteristics.

## 4 Discussion

Compared with previous aroma studies on other tea types or aroma profiles, the present work specifically clarifies the material basis of the honey and sweet potato-like aroma of WYBT and further links decisive odorants with olfactory receptor interactions through integrated docking and MD simulations. Despite constituting merely 0.01% of tea's dry weight, volatile compounds are critical determinants of tea quality and directly shape consumers' overall sensory experience (Ho et al., 2015). Among the diverse aroma profiles of WYBT, the honey and sweet potato-like aroma stands out as a significant characteristic (Li et al., 2024). It is important to emphasize that no individual compound was identified as solely responsible for the complete honey and sweet-potato-like aroma. Rather, this distinctive aroma profile results from the complex balance and collective interaction of multiple volatile compounds, aligning with existing studies on sweet potato aroma composition (Abugu et al., 2025).

From a compositional perspective, the aroma profile of WYBT was mainly shaped by terpenes, alcohols, aldehydes, esters, and ketones. This result is in line with previous studies on black tea aroma, especially reports on sweet potato- and honey-like aroma styles, in which terpenoids and aldehydes were regarded as major contributors to characteristic sweet, floral, fruity, and honey-like notes (Zhai et al., 2022; Yao et al., 2024; Chen et al., 2025). its characteristic aroma is likely associated with the convergence of multiple formation pathways, particularly carotenoid degradation, Strecker degradation, and Maillard-related reactions. In this context, the co-occurrence of carotenoid-derived volatiles such as *β*-damascenone, *α*-ionone, and *β*-cyclocitral, together with Strecker- and Maillard-related compounds such as benzeneacetaldehyde and 2-amylfuran, indicates that the honey- and sweet potato-like aroma of WYBT is better understood as a pathway-integrated aroma system rather than a single-class aroma phenomenon (Ho et al., 2015; Yao et al., 2024).

More importantly, five compounds, namely *β*-damascenone, benzeneacetaldehyde, *α*-ionone, *β*-cyclocitral, and 2-amylfuran, were identified as the decisive odorants for the characteristic aroma of WYBT. Among them, *β*-damascenone and *α*-ionone are carotenoid-derived compounds with very low odor thresholds and well-recognized floral-sweet sensory properties, which explains their strong contribution to the aroma quality of black tea even at relatively low concentrations (Chen et al., 2022). Benzeneacetaldehyde, a Strecker degradation product of phenylalanine, is closely associated with sweet and honey-like notes and has also been reported as a key contributor in several black tea aroma studies (Yao et al., 2023; Yin et al., 2022; Zheng et al., 2023). *β*-Cyclocitral, another carotenoid-derived volatile, and 2-amylfuran, which is associated with Maillard and Strecker-related reactions, further contribute fruity, sweet, and roasted nuances that enrich the sweet potato-like sensory character of WYBT (Xiao et al., 2022; Yang et al., 2022). At the molecular level, docking and MD simulations further suggested that these five decisive odorants can interact stably with representative olfactory receptor models through hydrophobic interactions and, in some cases, hydrogen bonding. Although these simulations do not directly prove in vivo olfactory perception, they provide structural support for why these compounds may play prominent roles in aroma recognition. Taken together, these findings indicate that the honey- and sweet potato-like aroma of WYBT is an emergent sensory outcome of multi-odorant synergy, in which the chemical abundance of key volatiles, their sensory effectiveness, and their receptor-binding potential jointly shape the final aroma perception.

Notably, these five decisive odorants have also been repeatedly reported in sweet potato aroma studies, particularly in raw, boiled, and baked sweet potato products, where they are considered important contributors to characteristic sweet, roasted, and potato-like notes (Abugu et al., 2025; Shen et al., 2024; Zhang et al., 2025). This cross-system consistency provides additional support for the sensory relevance of these compounds in WYBT and helps explain why the aroma of this tea shares recognizable similarity with sweet potato-related flavor perceptions. Therefore, the characteristic aroma of WYBT may be understood as the outcome of overlapping volatile signatures from black tea aroma chemistry and sweet potato-like sensory drivers. In this context, the present study not only identifies the decisive odorants underlying the target aroma profile, but also advances current understanding by linking chemical screening, sensory validation, and receptor-level interaction analysis into an integrated mechanistic framework.

Thus, the present study advances previous work in two ways. First, it moves beyond descriptive volatile profiling by establishing a stepwise evidence chain from volatile detection to sensory confirmation of decisive odorants. Second, it extends mechanistic interpretation from the sensory and chemical levels to the receptor-interaction level through docking and MD simulations. Although these simulations do not directly prove in vivo olfactory perception, they provide additional structural support for why the identified odorants may play prominent roles in aroma recognition. Taken together, these findings offer a more integrated explanation of WYBT aroma formation and perception, and they provide a stronger mechanistic basis for understanding the distinctive honey and sweet potato-like aroma of this tea.

## 5 Conclusion

This study systematically elucidated the key odorants underlying the honey- and sweet potato-like aroma of WYBT by integrating molecular sensory analysis with computational simulation. A total of 323 volatile compounds were identified, from which 19 key odor-active compounds were retained through integrated chemical and olfactory evaluation. Sensory correlation analysis, aroma recombination, and omission experiments further demonstrated that *β*-damascenone, benzeneacetaldehyde, *α*-ionone, *β*-cyclocitral, and 2-amylfuran are the decisive odorants shaping the characteristic aroma of WYBT. Molecular docking and MD simulations suggested that these compounds could interact stably with representative olfactory receptor models mainly through hydrophobic interactions and hydrogen bonding, providing additional structural support for their sensory importance. Overall, the present study not only clarifies the material basis of the distinctive honey- and sweet potato-like aroma of WYBT, but also establishes an integrated framework linking volatile composition, sensory contribution, and receptor-level interaction. These findings provide a more mechanistic understanding of aroma formation and perception in WYBT and offer a theoretical basis for quality evaluation and process optimization of this unique tea product.

## CRediT authorship contribution statement

**Zhichao Lin:** Writing – original draft, Visualization, Investigation, Formal analysis, Data curation, Conceptualization. **Mengzhen Xia:** Writing – original draft, Validation, Investigation, Formal analysis, Data curation. **Li Niu:** Project administration, Methodology, Funding acquisition, Conceptualization. **Guohe Chen:** Validation, Methodology, Investigation, Conceptualization. **Lianqing Wang:** Writing – review & editing, Validation, Methodology, Investigation. **Jianan Huang:** Supervision, Project administration, Funding acquisition, Conceptualization. **Zhonghua Liu:** Supervision, Resources, Project administration, Funding acquisition, Conceptualization. **Chao Wang:** Writing – review & editing, Supervision, Project administration, Methodology, Funding acquisition, Formal analysis, Data curation.

## Ethical statement

The authors ensure that the work described has been carried out in accordance with The Code of Ethics of the World Medical Association (Declaration of Helsinki) for experiments involving humans. The ethical approval of sensory evaluation is not required by national laws. No human ethics committee or formal documentation process is available for sensory evaluation. The authors confirm that the appropriate protocols for protecting the rights and privacy of all participants were utilized during the execution of the research, including no coercion to participate, full disclosure of study requirements and risks, verbal consent of participants, no release of participant data without their knowledge, and the ability to withdraw from the study at any time.

## Declaration of competing interest

The authors declare that they have no known competing financial interests or personal relationships that could have appeared to influence the work reported in this paper.

## Data Availability

Data will be made available on request.
