# Supplementary material for: Elucidation of the key odorants contributing to the distinctive honey and sweet potato-like aroma in Wuyi black tea
Source: Food Chem X. 2026 May 22;36:104022. doi: 10.1016/j.fochx.2026.104022 (PMC13223701; doi:10.1016/j.fochx.2026.104022)

**Figure caption:**

**Figure S1.** Correlation heatmap of five sensory aroma descriptors in six selected Wuyi black tea (WYBT) samples. The heatmap illustrates the pairwise relationships among floral, fruity, honey-like, sweet potato-like, and woody attributes. Red and blue indicate positive and negative correlations, respectively, and color intensity reflects the strength of the correlation. Asterisks indicate significant correlations (* p < 0.05, ** p < 0.01).

Figure S1.


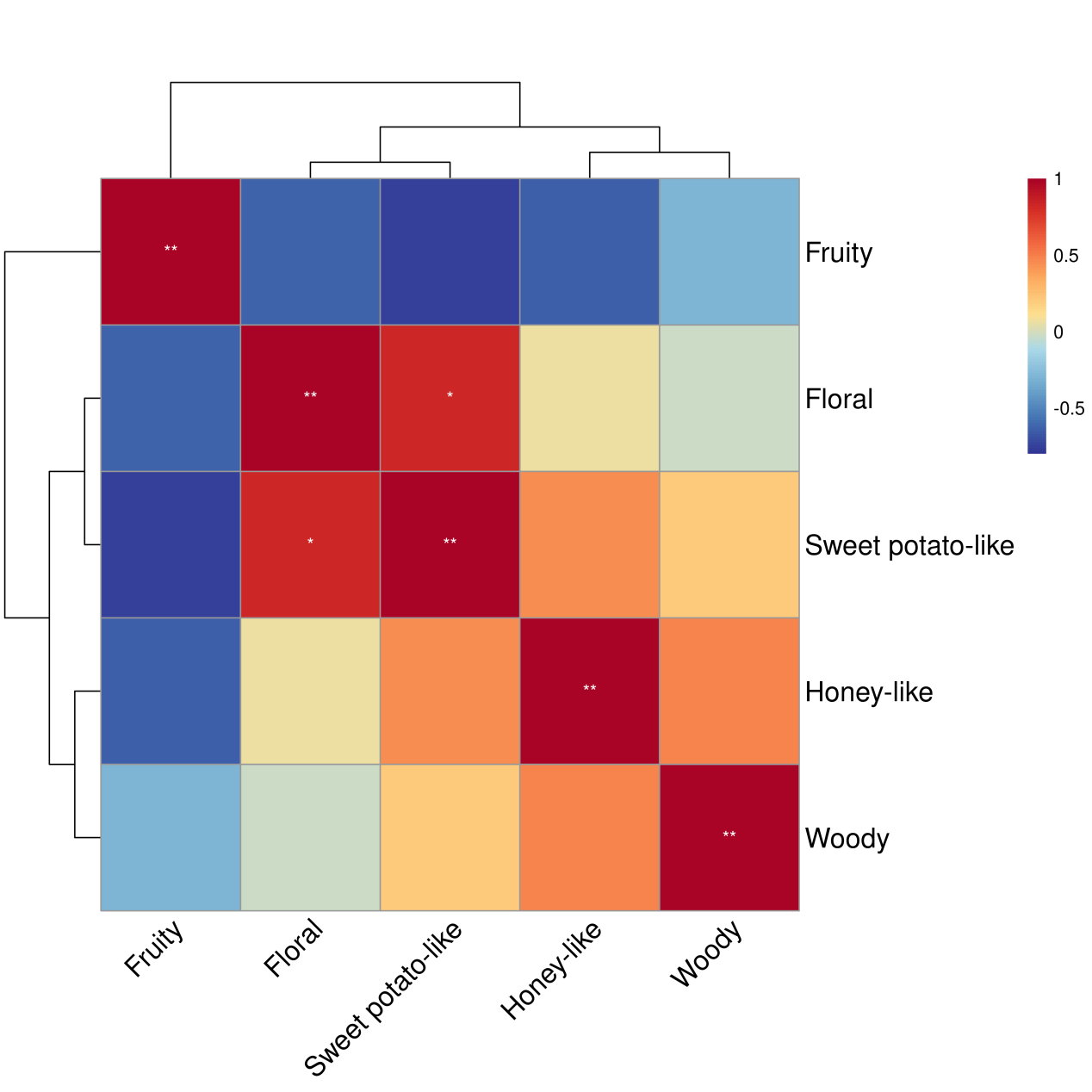

Supplement: Supplementary material 1 [file mmc1.docx]
